# The Performance Scales disability measure for multiple sclerosis: use and sensitivity to clinically important differences

**DOI:** 10.1186/s12955-017-0614-z

**Published:** 2017-03-09

**Authors:** Carolyn E. Schwartz, Victoria E. Powell

**Affiliations:** 1 DeltaQuest Foundation, Inc., 31 Mitchell Road, Concord, MA 01742 USA; 2 Departments of Medicine and Orthopaedic Surgery, Tufts University Medical School, Boston, MA USA

**Keywords:** Patient-reported outcomes, Multiple sclerosis, Disability, Quality of life, Clinical trials outcomes, Rehabilitation, Epidemiological research, Performance Scales, Review, Responsiveness, Interpretation

## Abstract

**Background:**

In 1993, the Performance Scales© was created to assess multi-dimensional disability in multiple sclerosis (MS). This tool has been used in a variety of settings and study designs internationally. The present work provides an overview of the history and psychometric characteristics of the Performance Scales©, reviews its use over the past two decades, and summarizes its responsiveness to subgroup differences.

**Methods:**

A Google Scholar and Ovid search yielded 230 articles citing the Performance Scales©, of which 82 studies used the tool in empirical research. Twelve articles provided sufficient information to enable computation of effect sizes. Forest plots were used to show effect sizes for the overall summary score and by domain by patient demographics, MS disease trajectory, and treatment adherence.

**Results:**

The Performance Scales© evidenced sensitivity to clinically important differences by disease trajectory and age (for selected domains). In contrast, groups distinguished by patient adherence to disease-modifying therapies and ethnicity were relatively small.

**Conclusions:**

The Performance Scales© has been used in a large number of studies since its development, suggesting that this psychometrically sound tool is acknowledged to be a useful tool for MS clinical research. It is recommended that future work include the entire measure, so that the whole-person impact of MS can be characterized and considered in MS outcome research.

## Background

The use of patient-reported outcomes has become increasingly standard in multiple sclerosis (MS) clinical research and practice. While clinical exams can provide useful information about objective disability indicators (e.g., mobility difficulties), it is widely acknowledged that important aspects of treatment impact can only be addressed by asking the patient directly. For example, fatigue, numbness, and cognitive difficulties can impact an individual’s ability to engage in work and other important activities of daily living and they are not always visible to the ‘objective’ observer. In 1993, the Performance Scales© was created to assess multi-dimensional disability in MS. This tool has been used in a variety of settings and study designs internationally. Yet, recent reviews of MS outcome measures have neglected to mentioned or cite this measure [1, 2], despite its use in a substantial number of research studies. Its brevity, strong psychometric characteristics, and availability in many language translations render it useful in a number of clinical and research contexts. The present work provides an overview of the history and psychometric characteristics of the Performance Scales©, reviews its use over the past two decades, and summarizes its responsiveness to subgroup differences.

## History of the Performance Scales©

The development of the Performance Scales© was motivated by the need to have patient-reported indices of disability to provide assessment alternatives to the neurologic examination. The gold standard for assessing disability in MS is the Expanded Disability Status Scale (EDSS) [3], which is scored on the basis of clinician answers to the Functional Systems Scores (FSS) and requires substantial clinician time (about 40 min). The EDSS score is driven by ambulation disability and does not reflect other, important but hidden (i.e., not visible) aspects of disability sufficiently (e.g., cognitive symptoms). Additionally, the EDSS has well-documented issues with inter-rater reliability in the minimal-to-moderate range of disability. This means that two neurologists rating the same individual may vary in their scoring by one point on the ten-point scale [4, 5]. This one-point difference is the same as the standard definition of clinically significant change used in clinical trials [6]. Furthermore, the EDSS is differentially sensitive to change, depending on the initial disability level. Accordingly, a change of 1 point on the EDSS reflects more dramatic change at higher levels of disability. This reliability problem reduces the responsiveness of the EDSS as well as the statistical power of any study that uses the EDSS as an outcome.

In addition to the above psychometric concern of reliability, the Performance Scales© measure was created to consider more relevant domains of MS-specific disability. This focus on content validity was stimulated by qualitative research with MS patients in the context of developing a psychosocial intervention for patients and caregivers [7]. Elicited patient description of their disability and its impact on their life did not correspond to their healthcare providers’ descriptions of their disability [8]. The patients’ answers reflected a wide range of domains that were not reflected by the EDSS or FSS, or by generic health status measures, such as the widely used SF-36™. Hence it was clear that there was a need for a robust measure of MS disability that covered the full multi-dimensional territory affected.

## Psychometric characteristics of the Performance Scales©

### Domains

The Performance Scales© includes the following eight domains: Mobility, Hand Function, Vision, Fatigue, Cognitive, Bladder/Bowel, Sensory, and Spasticity Symptoms.

### Format and response options

Each domain scale begins with a brief definition of the domain, and then asks the respondent to endorse what level of disability s/he experiences with regard to performing his/her normal activities of daily living. The estimated completion time of 2 min. Higher scores represent more impairment.

### Validation studies of the Performance Scales©

The initial validation of the Performance Scales© was a multi-site, cross sectional study implemented with 13 MS centers around the United States and Canada that was completed in 1997 [9]. The Schwartz et al. [9] study included 274 MS patients and 296 healthy controls [9]. The study evaluated the reliability and validity of the Performance Scales© in comparison to clinical measures, and other patient-reported outcomes. Subsequent work by Marrie and Goldman [10] evaluated the Performance Scales© criterion and construct validity in 44 people with MS, and work by Motl and Snook [11] evaluated its discriminant and convergent validity in 133 people with MS.

## Reliability

### Stability

The test-retest reliability coefficient for the total score of the Performance Scales© was 0.89, and the coefficients for the eight domain scores ranged from 0.65 to 0.91. The Mobility domain score had the highest test-retest reliability, and the Sensory domain score had the lowest test-retest reliability.

### Internal consistency

Because each domain score has only one score, the internal consistency of the Performance Scales© domain scores was not evaluated. The alpha reliability of the total score for the Performance Scales© was determined to be 0.78.

## Validity

### Construct validity

The Performance Scales© summary score is associated in the expected direction with other clinical and self-reported measures of disability. Using Cohen’s [12] criteria for small (*r =* 0.1), medium (*r =* 0.3), and large (*r =* 0.5) correlation coefficients, we found that the Performance Scales© summary score had a small correlation with disease duration, and large correlations with the disease-specific and generic measures of functional status. Motl and colleagues confirmed large correlations with disease-specific measures, reported moderate correlations with the performance-based accelerometer [13], and small correlations with self-reported exercise.

### Discriminant validity

In our original validation study, we found that the Performance Scales© summary score is able to distinguish the following known groups: healthy control subjects; minimally, moderately, and severely disabled MS patients [9]. Remarkably similar mean values by level of EDSS disability were reported in an independent replication study by Motl and colleagues [14]. Unlike the generic functional health measures included in the study, the Performance Scales© means were in the expected order (i.e., lowest to highest), whereas the generic health measures yielded an incorrect order with respect to the EDSS groupings. This finding is consistent with past research that has documented that disease-targeted scales provide unique information not captured by generic measures [15], and thus may be more sensitive to higher levels of disability.

### Incremental validity of the Performance Scales

The Performance Scales© tool assesses self-reported disability. Its overlap with the Symptom Inventory (SI) measure of impairment is relatively high among minimally and moderately disabled people with MS (r = 0.80) but substantially lower among severely disabled people with MS (r = 0.60 for the SI long form, and 0.48 for the SI short form). This finding suggests that the two measures might have more incremental validity among severely disabled patients. Of note, the Performance Scales© summary scores explained 41% of the variance in predicting utility scores; 38% of the variance in predicting the clinician-rated EDSS; 36% of the variance in predicting the clinician-rated Disease Steps; and 30% of the variance in predicting the clinician-rated Ambulation Index. Motl and colleagues replicated these same incremental validity analyses in an independent sample of 129 MS patients [14]. These analyses suggest that the Performance Scales© summary score measures an outcome that is distinct and complementary to existing clinical, MS-specific, and generic self-report measures.

## Scoring and alternative uses of the Performance Scales©

The standard scoring algorithm for the Performance Scales© is a simple ordinal summary index. Factor analyses support its unidimensionality, and confirm a very high correlation between a simple sum and a weighted sum based on factor loadings [16]. It is also possible to use the individual domain scores to describe change over time. This use of the Performance Scales© has been tested and validated in a number of studies using MS patient registry data [10]. This approach may be useful in a clinical setting to identify areas of focus for clinical intervention. The caveat to this use of individual domain scores is that it may lead to ignoring the multidimensional causes of disability in MS, and thus render the scores less responsive and relevant.

## Available translations of the Performance Scales©

There are currently 20 translations of the Performance Scales©, created using the standard methods recommended by large clinical trial collaborative groups and other organizations actively involved in culturally-equivalent translations of patient-reported outcomes [17–20]. The availability of so many translations makes this patient-reported outcome tool particularly feasible for international collaborations.

The present work sought to investigate how the Performance Scales© has been used since its inception, and to estimate the tool’s responsiveness to clinically important change.

## Methods

A Google Scholar search was done searching all published articles referencing the Schwartz et al. [9] and Marrie and Goldman [10] articles through June 3, 2016, since these two articles were the most direct validation studies of the tool. Additionally an Ovid search was done using Medline only and the term “Performance Scales”. It was followed by a second search using all Ovid databases and the terms “Performance Scales” and “multiple sclerosis.” Both authors then reviewed each article according to the following inclusion and exclusion criteria. Articles were included for further consideration if they actually used the Performance Scales© in the reported study. Articles were excluded if they were a review, book, or thesis; did not use the Performance Scales© in the reported study; were not in English; were a copyright infringement; or we were not able to procure a copy of the article despite multiple efforts. From those citations that presented empirical data, we identified the papers that provided means for patient subgroups or study groups. This information was then used to calculate an effect size using Cohen’s D [12] as follows: $d=\frac{M_1 - {M}_2}{S{ D}_{pooled}}$ for means with independent samples. Forest plots showing the effect sizes were generated to show effect size by patient demographics, MS disease trajectory, and treatment adherence.

All analyses were done using Microsoft Office Excel 2010 and Stata 13 [21].

## Results

**Fig. 1:**
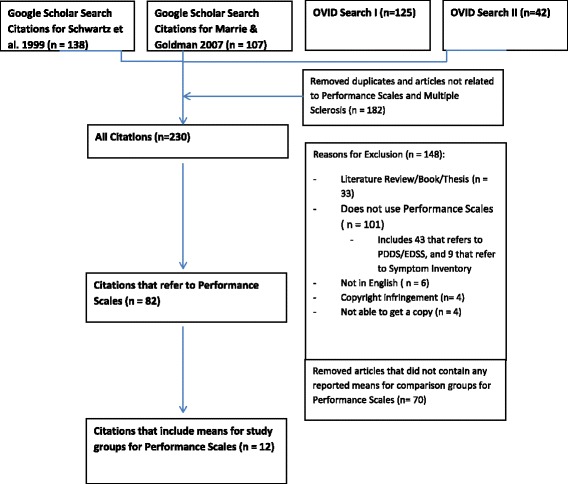
Exclusion tree for articles that were identified in the Ovid search

Figure 1 shows the exclusion tree for articles that were identified in the Google Scholar and both Ovid searches. After removing duplicate articles as well as articles that were not referring to the Performance Scales© (many articles used “performance scales” as a general term), 230 citations remained, Of the 230 citations, 148 were excluded because they were not empirical studies, referred to a different tool used and/or validated in the original Schwartz et al. [20] study, did not use the Performance Scales©, were not in English, or were a copyright infringement. From this subset of 82 articles that did use the Performance Scales©, only 12 articles provided sufficient information to enable computation of effect sizes.

Table 1 and the Appendix provide further information about the 82 studies using the Performance Scales© on over 321,000 people with MS.^1^ About 73% of the studies were observational, 21% were measurement validation, and 6% were intervention studies. The majority of studies used cross-sectional designs, and about one third were longitudinal. Of the longitudinal studies, only one included more than two timepoints. About half of the studies utilized data from the North American Research Committee on MS (NARCOMS) registry, and the remaining studies used data from the Veterans Health Administration, local chapters of the National MS Society, and MS clinics based at hospitals or universities. Funding sources for the studies were dominated by foundation funding, followed by federal grants, industry grants, and unspecified or internal funding.

**Table 1:** Summary of studies using the Performance Scales© 1999-2016

	All citations (*n* = 82)		Forest plots citations (*n* = 12)	
Study Type	n	%	n	%
Observational	60	73%	11	92%
Intervention	5	6%	1	8%
Measurement Validation	17	21%	0	0%
Design Type				
Cross Sectional	51	62%	6	50%
Longitudinal	29	35%	6	50%
Randomized Controlled Trial	2	2%	0	0%
Participant Source				
NARCOMS	43	52%	7	58%
Vetrans Health Administration	10	12%	1	8%
Midwest Chapters, National MS Society	5	6%	0	0%
Cleveland Clinic Mellen Center	9	11%	2	17%
Other	15	18%	2	17%
Funding Source^a^				
Internal/Academic	8	10%	2	17%
Industry	21	26%	4	33%
Federal	25	30%	2	17%
Foundation	51	62%	8	67%
Not Specified	11	13%	0	0%

^a^Studies may have more than 1 funding source

**Fig. 2:**
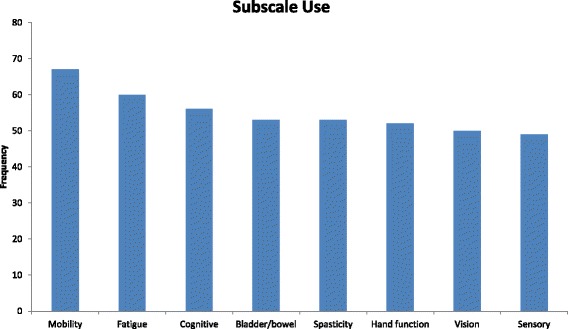
Bar chart showing how often each of the Performance Scales© domain scores were used throughout the reviewed studies

Of the 82 articles that used the Performance Scales©, 52 used the measure as an outcome, and 30 used it either as a predictor or to classify their sample. The majority of studies used the measure to define disability status or used it as a general health outcome. While the majority of studies used the full measure, there were a number that used either one or some of the domain scores. In total, 47 studies used all 8 of the Performance Scales© domain scores, 21 studies used only one domain score, and 14 used between 2 and 7 domain scores. The most commonly used domain score was the Mobility domain score which was used in 67 studies, followed by Fatigue which was used in 60 studies. Figure 2 shows the number of studies that used each of the 8 domain scores.

**Fig. 3:**
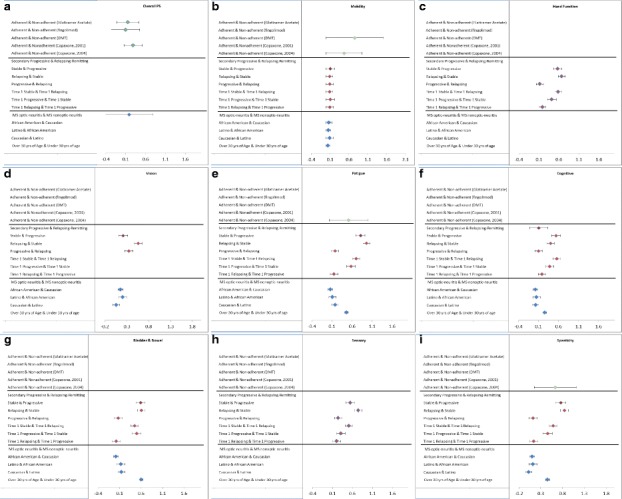
**a**-**i** Effect sizes with 95% confidence interval bars by subgrouping variable and domain, as available

Figures 3a-l show the effect sizes with 95% confidence interval bars by subgrouping variable and domain, as available. In comparisons of adherent versus non-adherent patients, the Performance Scales© Summary Score detected a small effect size difference, whereas the Mobility and Fatigue effect size differences were moderate (i.e., clinically significant). In comparisons by disease trajectory, small effect size differences were detected by the Mobility and Cognitive domains; moderate effect size differences were detected by the Bladder/Bowel, Hand Function, Sensory, and Vision domains; and large effect size differences were detected by the Fatigue and Spasticity domains. Effect sizes varied, however, for each domain score by specific comparison. In ethnicity comparisons, detected effect size differences were small across all domain scores. In comparisons by age group (<=30 years versus > 30), effect size differences were small for Cognitive, Depression, Mobility, and Tremor/Coordination; but moderate for Fatigue and Bladder/Bowel.

## Discussion

The Performance Scales© has been used in a large number of studies since its development, suggesting that this psychometrically sound tool is acknowledged to be a useful and reliable tool for MS clinical research. Our results support the tool’s sensitivity to clinically important differences by disease trajectory and age (for selected domains). Based on Cohen’s effect size interpretation guidelines [12], the Performance Scales© Summary score is able to detect small differences between groups, and thus could be used in studies comparing the effectiveness of disease-modifying therapies for MS. The individual domain scores demonstrated sensitivity to small, moderate, and large group differences; depending on the study research question and comparison group. We would thus recommend collecting all eight domain scores to maximize the likelihood of detecting small differences, and being able to characterize the domain most effected by having data on all the individual domain scores. In contrast, groups distinguished by patient adherence to disease-modifying therapies and ethnicity were relatively small.

The multidimensional format of the Performance Scales© can be particularly useful for MS outcomes research because it allows clinical researchers to characterize the *profile* of changes within and across individuals. Illustrated by the range of effect sizes detected across domains and by grouping variable, our review provides useful information for planning future studies. For example, if an investigator wishes to design a study with sufficient power to compare disability levels across groups as a function of treatment adherence, then the sample sizes will need to be on the order of about 300 per group [8]. This will enable adequate statistical power given a Type I error rate of 0.05 . In contrast, if disease trajectory or age-related differences are the primary focus, then the study can include only about 62 patients per group to have similar power and Type I error rate. Thus, the comparisons shown in the Forest Plots included herein can assist investigators in planning future studies using the Performance Scales©.

The limitations of this work include the fact that relatively few of the published empirical studies provided data that allowed for their inclusion in the effect size computations. We are thus only able to comment on the tool’s responsiveness in a small subsample of the literature. Further, information on sex and age distributions was limited. Future research might address how sex and age distributions differ in studies using the Performance Scales© or other disease-specific patient-reported outcomes.

It is unfortunate that so few studies used and/or reported the Performance Scales© Summary Score. Of the 82 studies, 35 are using only one or some of the domain scores, rather than the whole multidimensional measure as it was intended. It is recommended that future work include the entire measure, so that the whole-person impact of MS can be characterized and considered in MS outcome research.

## Conclusion

The Performance Scales© has been used in a large number of studies since its development. The tool is sensitive to small changes over time in overall MS disability, and to moderate and small changes over time in selected domains. This responsiveness to clinically relevant change as well as its well-documented strong psychometric characteristics make it a useful tool for MS clinical research. lt is recommended that future work include the entire measure, so that the whole-person impact of MS can be characterized and considered in MS outcome research.

## Appendix

Studies utilizing the Performance Scales© 1999–2015.

## Abbreviations

EDSS: Expanded Disability Status Scale
FSS: Functional Systems Scores
MS: Multiple sclerosis
SI: Symptom Inventory

## Footnotes

1 Since many studies use NARCOMS samples or subsamples, it is not possible to know how many of the 321,000 people are actually the same person in multiple studies. Data bases provided to NARCOMS investigators are de-identified.

## References

[1] Cohen ET, Potter K, Allen DD, Bennett SE, Brandfass KG, Widener GL (2015). Selecting rehabilitation outcome measures for persons with multiple sclerosis. Int J MS Care.

[2] Park C, Watson C (2015). Patient-reported outcome (PRO) measures used in secondary-progressive multiple sclerosis (SPMS) studies: A Systematic Review (P3. 215). Neurology.

[3] Kurtzke JF (1983). Rating neurologic impairment in multiple sclerosis: an expanded disability status scale (EDSS). Neurology.

[4] Pia Amato M, Fratiglioni L, Groppi C, Siracusa G, Amaducci L (1988). Interrater reliability in assessing functional systems and disability on the Kurtzke scale in multiple sclerosis. Arch Neurol.

[5] Noseworthy JH, Vandervoort MK, Wong CJ, Ebers GC, Group CCMS (1990). Interrater variability with the Expanded Disability Status Scale (EDSS) and functional systems (FS) in a multiple sclerosis clinical trial. Neurology.

[6] Weiner HL, Dau PC, Khatri BO, Petajan JH, Birnbaum G, McQuillen MP (1989). Double-blind study of true vs. sham plasma exchange in patients treated with immunosuppression for acute attacks of multiple sclerosis. Neurology.

[7] Schwartz CE, Rogers M (1994). Designing a psychosocial intervention to teach coping flexibility. Rehabil Psychol.

[8] Schwartz CE, Fierston S (1995). The two sides of pseudo-bulbar disorder in multiple sclerosis: comparing one patient’s experience with a review of the neurologic literature. Neurorehabilitation.

[9] Schwartz CE, Vollmer T, Lee H (1999). Reliability and validity of two self-report measures of impairment and disability for MS. North American Research Consortium on Multiple Sclerosis Outcomes Study Group. Neurology.

[10] Marrie RA, Goldman M (2007). Validity of performance scales for disability assessment in multiple sclerosis. Mult Scler J.

[11] Motl RW, Snook EM (2008). Confirmation and extension of the validity of the Multiple Sclerosis Walking Scale-12 (MSWS-12). J Neurol Sci.

[12] Cohen J (1992). A power primer. Psychol Bull.

[13] Motl RW, Pilutti L, Sandroff BM, Dlugonski D, Sosnoff JJ, Pula JH (2013). Accelerometry as a measure of walking behavior in multiple sclerosis. Acta Neurol Scand.

[14] Motl RW, Schwartz CE, Vollmer T. Continued validation of the Symptom Inventory in multiple sclerosis. Journal of Neurological Sciences. 2009:in press. 10.1016/j.jns.2009.06.015 19592041

[15] Vickrey BG, Hays RD, Genovese BJ, Myers LW, Ellison GW (1997). Comparison of a generic to disease-targeted health-related quality-of-life measures for multiple sclerosis. J Clin Epidemiol.

[16] Nunnally JC, Bernstein IH (1994). Psychometric Theory.

[17] DeWolf L, Koller M, Velikova G, Johnson C, Scott N, Bottomley A (2009). EORTC Quality of Life Group Translation Procedure.

[18] Aaronson NK, Acquadro C, Alonso J, Apolone G, Bucquet D, Bullinger M (1992). International Quality of Life Assessment (IQOLA) Project. QualLife Res.

[19] Hawkins M, Osborne R (2010). Health Literacy Questionnaire: Translation and cultural adaptation procedure.

[20] Hunt SM, Alonso J, Bucquet D, Niero M, Wiklund I, McKenna S (1991). Cross-cultural adaptation of health measures. European Group for Health Management and Quality of Life Assessment. Health Policy.

[21] StataCorp, Software S (2013). Stata: Release 13.

